# Layered inflammation in pacemaker infection: histopathologic correlation with metabolic imaging

**DOI:** 10.1093/ehjcr/ytag011

**Published:** 2026-01-09

**Authors:** Yoshiko Eto, Nobuhiro Tahara, Yoshihiro Fukumoto

**Affiliations:** Division of Cardiovascular Medicine, Department of Medicine, Kurume University School of Medicine, 67 Asahi-machi, Kurume 830-0011, Japan; Division of Cardiovascular Medicine, Department of Medicine, Kurume University School of Medicine, 67 Asahi-machi, Kurume 830-0011, Japan; Division of Cardiovascular Medicine, Department of Medicine, Kurume University School of Medicine, 67 Asahi-machi, Kurume 830-0011, Japan

**Figure:**
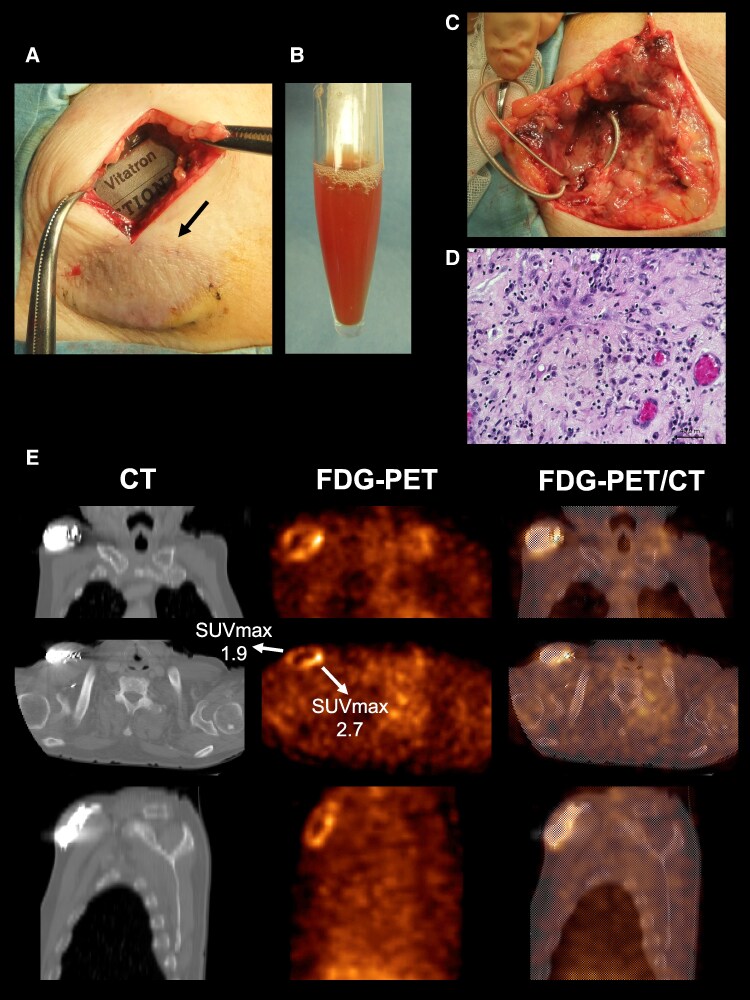


A 77-year-old man presented with progressive brown discolouration and swelling over his pacemaker pocket (*Panel A*, arrow), 8 years after dual-chamber pacemaker implantation for advanced atrioventricular block. He remained afebrile with mild systemic inflammatory activity (C-reactive protein 0.69 mg/L, erythrocyte sedimentation rate 37 mm/h, and white blood cell count 5000/μL), and repeated blood cultures were negative. Transthoracic echocardiography revealed no vegetations or lead-associated masses. Given the inconclusive conventional findings suggestive of cardiac implantable electronic device (CIED) infection, 18F-fluorodeoxyglucose positron emission tomography combined with computed tomography (FDG-PET/CT) was performed, revealing a spatial gradient of FDG uptake around the generator pocket (*Panel E*). The maximum standardized uptake value (SUVmax) increased from 1.9 in the superficial layer to 2.7 in the posterior perigenerator tissue directly adjacent to the generator body, particularly around the header–lead junction, indicating higher inflammatory activity within the FDG uptake region. Complete system extraction yielded purulent material with polymicrobial growth (*Staphylococcus aureus*, *Escherichia coli*, and *Enterococcus* species; *Panel B*). Histopathological examination of excised granulation tissue revealed mixed inflammatory infiltrates composed of neutrophils, lymphocytes, and macrophages (*Panels C and D*). Quantitative analysis demonstrated a 42% increase in inflammatory cell density (188 vs. 248 cells per high-power field), closely paralleling the rise in SUVmax from 1.9 to 2.7, thereby establishing a direct correlation between metabolic and histologic inflammatory activity. Following targeted antibiotic therapy and complete device extraction, the patient achieved full recovery, and epicardial pacing leads with an abdominal generator were implanted.

This case provides novel histopathologic evidence of spatially graded FDG uptake intensity in a CIED infection. While previous reports established FDG-PET/CT as a sensitive modality for diagnosing device infection, the quantitative correspondence between metabolic signal intensity and inflammatory cell burden has not been demonstrated. These findings support the usefulness of FDG-PET/CT for identifying localized infection activity, even in patients with minimal systemic inflammation or negative blood cultures.

## Data Availability

The data underlying this article will be shared on reasonable request to the corresponding author.

